# Transformation from slip to plastic flow deformation mechanism during tensile deformation of zirconium nanocontacts

**DOI:** 10.1038/srep42901

**Published:** 2017-02-20

**Authors:** Kohei Yamada, Tokushi Kizuka

**Affiliations:** 1 Division of Materials Science, Faculty of Pure and Applied Sciences, University of Tsukuba, Tsukuba, Ibaraki 305-8573, Japan

## Abstract

Various types of nanometer-sized structures have been applied to advanced functional and structural devices. Inherent structures, thermal stability, and properties of such nanostructures are emphasized when their size is decreased to several nanometers, especially, to several atoms. In this study, we observed the atomistic tensile deformation process of zirconium nanocontacts, which are typical nanostructures used in connection of nanometer-sized wires, transistors, and diodes, memory devices, and sensors, by *in situ* transmission electron microscopy. It was found that the contact was deformed via a plastic flow mechanism, which differs from the slip on lattice planes frequently observed in metals, and that the crystallinity became disordered. The various irregular relaxed structures formed during the deformation process affected the conductance.

As the size of metals decreases to the nanometer scale, dislocation motion is suppressed, or even absent, while applying external forces. When the grain size of polycrystalline metals is decreased to 10–100 nm, grain boundary sliding and grain rotation govern deformation instead of dislocation-mediated slip^1^,^2^,^3^,^4^,^5^,^6^,^7^. In particular, as the width of deformation region is reduced to several atoms, the deformation mechanism is transformed from dislocation-mediated slip to non-slip manners, i.e., homogeneous slip^8^,^9^,^10^,^11^,^12^. After the transformation of deformation mechanics, the critical shear stress of nanometer-size metals increases by several tens of times or more in comparison with that of dislocation-mediated slip, e.g., ~1 GPa for silver^11^ (Ag) and ~5 GPa for rhodium^12^ (Rh). Such a transformation is observed in metallic nanocontacts (NCs) with face-centered cubic (fcc) structures^10^,^11^,^12^. Since the number of primary slip systems in fcc structure metals is 12, slip along equivalent slip systems can occur easily^13^; deformation via slip continues until the size is reduced to less than several nanometers and subsequent fracture occurs, regardless of whether the dislocation is mediated. Conversely, the primary slip system of metals with hexagonal-closed packed (hcp) structure is limited to only three equivalent systems on basal planes^13^. In addition, the rotation of the deformation regions of NCs is suppressed since the region is fixed by two adjacent rigid tips^11^. Atomic-size contacts of zinc (Zn) with an hcp structure show unstable melting-like behavior, related to different break conductance features from NCs of other metals^14^. Theoretical treatments, such as molecular dynamics (MD) simulations, show that the atomic configuration of the deformation region becomes disordered during the deformation in NCs with width less than several atoms; the deformation proceeds while the crystal structure crumbles and all atoms in the deformation region move simultaneously in a way like atomic motion in liquid^9^,^15^,^16^,^17^,^18^,^19^,^20^,^21^,^22^,^23^,^24^,^25^,^26^. Thus, atomistic observation of the deformation process of hcp-structure metal NCs is expected to reveal new deformation mechanics. In this paper, we focus on zirconium (Zr) NCs with this goal in mind.

## Results

**Figure 1:**
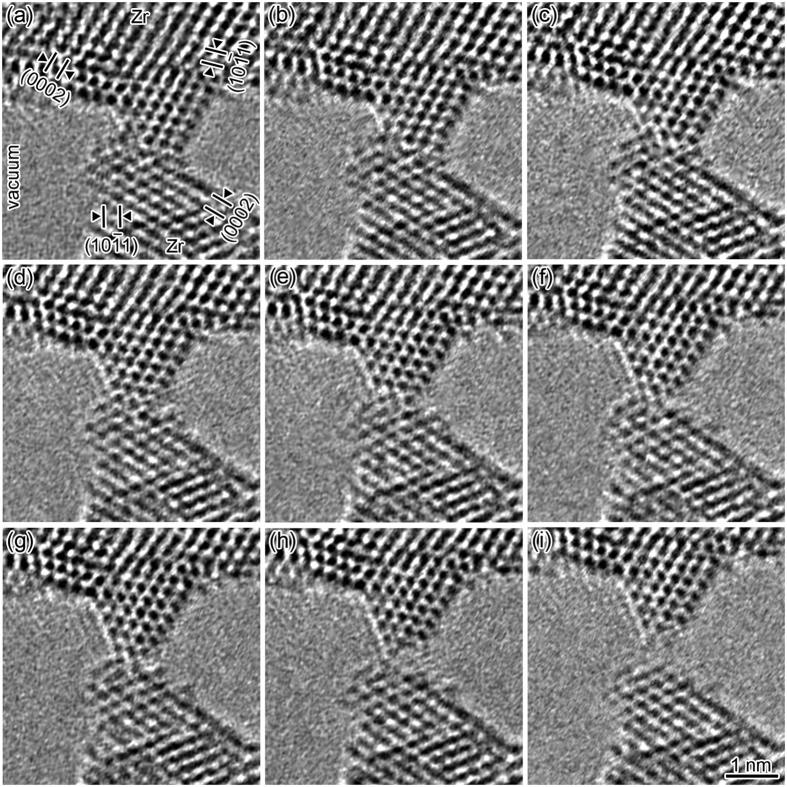
Time series of high-resolution images of Zr NC tensile deformation. The NC was elongated along the direction indicated by the arrow in Fig. 1a. The minimum cross-sectional width for each image is five atoms [(**a**), (**b**)], four atoms [(**c**)], three atoms [(**d**), (**e**)], two atoms [(**f**), (**g**)], and one atom [(**h**)]. (**i**) shows fracture. (See Supplementary Movie 1.) The average strain rate was 1.4 × 10^−2^/s.

**Figure 2:**
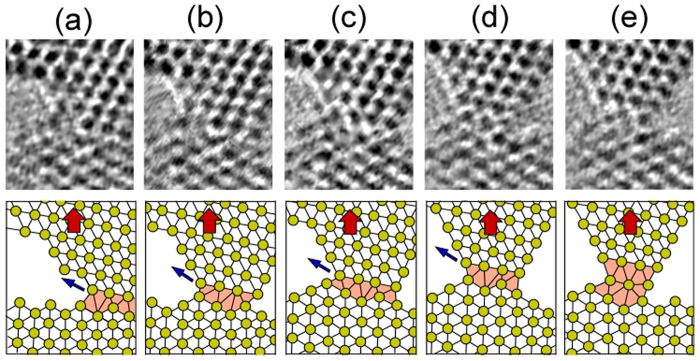
Time series of the enlarged high-resolution images of slip region in Fig. 1(a)–(e) (the shaded regions) and the models of their atomic configuration. The direction of tensile force parallel to the upper direction is indicated by the bold arrows. The slip direction is indicated by the fine arrows, which are parallel to the (0001) basal plane of the lower nanotip and tilt away from the tensile direction.

**Figure 3:**
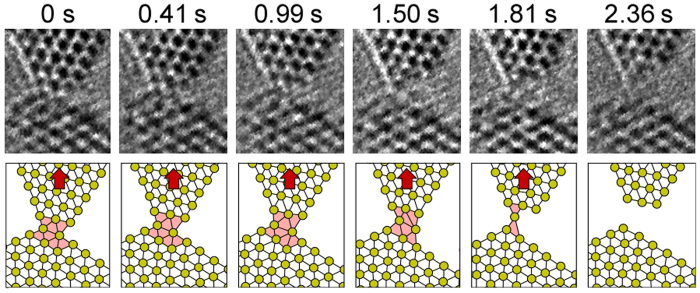
Time series of the enlarged high-resolution images of the deformation region from Fig. 1(f) and the models of their atomic configuration. The time of Fig. 1(f) is defined as 0 s for easy-to-understand. The direction of tensile force is indicated by the bold arrows, which are parallel to the expansion direction of the deformation region (the shaded regions). Note that the atomic configuration in the deformation regions differs from the hcp unit cell inside the nanotips.

Figure 1 shows the time series of high-resolution transmission electron microscopy (TEM) images of the tensile deformation process of a Zr NC (Supplementary Movie 1). Both the upper and lower dark regions in each image correspond to the Zr nanotips. The regions around the NC correspond to vacuum. The two types of lattice fringes observed on both nanotips correspond to the (0002) and (10
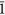
1) planes with hcp-structure Zr spacing of 0.26 nm and 0.25 nm, respectively. The incident-electron-beam direction, i.e., the observation direction of the NC, is parallel to the [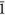
2
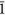
0] direction. From the lattice fringes it is found that the NC is a tilt grain boundary with a rotation angle of 90 ± 1°. First, the NC with a 5-atom-wide minimum cross section is shown in Fig. 1(a) and (b). When a tensile force acts on the NC, the upper nanotip slides on the (0001) plane of the lower nanotip, as indicated by the arrow in Fig. 1(a). Thus, slip occurs along the boundary plane. This slip corresponds to the typical slip system on the basal plane of an hcp structure [Fig. 1(a)–(e)]. Figure 2 shows the time series of the enlarged high-resolution images of the slip region in Fig. 1(a)–(e) and the models of their atomic configuration. The direction of the tensile force (the bold arrows in Fig. 2) is parallel to the upper direction, whereas the slip direction (the fine arrows in Fig. 2) is parallel to the (0001) basal plane of the lower nanotip, which tilts from the tensile direction. Thus, the slip direction tilts away from the tensile force direction. This deformation causes a decrease in contact width of five atoms [Fig. 2(c)], four atoms [Fig. 2(d)], three atoms [Fig. 2(e)], and two atoms [Fig. 2(f)]. Subsequently, the upper nanotip moves along the tensile force direction, i.e., the direction approximately perpendicular to the slip plane. Figure 3 shows the time series of the enlarged high-resolution images of the deformation region from Fig. 1(f) and the models of their atomic configuration (The time of Fig. 1(f) is defined as 0 s for easy-to-understand). The constriction region (the shaded regions in Fig. 3) expands along the direction parallel to the direction of tensile force (the bold arrows in Fig. 3). By this expansion, the interatomic distance in the deformation region is elongated and the bond angles become different from that inside the nanotips; the atomic configuration in the deformation region crumbles from the hcp unit cell. Thus, we found clearly that this deformation is non-slip deformation. Note that although the two types of lattice fringes observed clearly on both nanotips beside the minimum cross section, the lattice image of this region became blurred (see Supplementary Movie 1), indicating that the structure in the deformation region becomes disordered. Since stress does not become concentrated on a certain atomic plane during this deformation, atoms in the contact regions show diffusive motion similar to that in liquids although the contact is solid. Hence, we found that the deformation in NCs proceeds in a plastic flow manner. During the deformation, the contact width decreases from two atoms [Fig. 1(g)] to one atom [Fig. 1(h)]. The contact eventually fractures [Fig. 1(i)]. The observation reveals that, as the contact width decreases, the deformation mechanism transforms from slip on lattice planes to plastic flow deformation.

**Figure 4:**
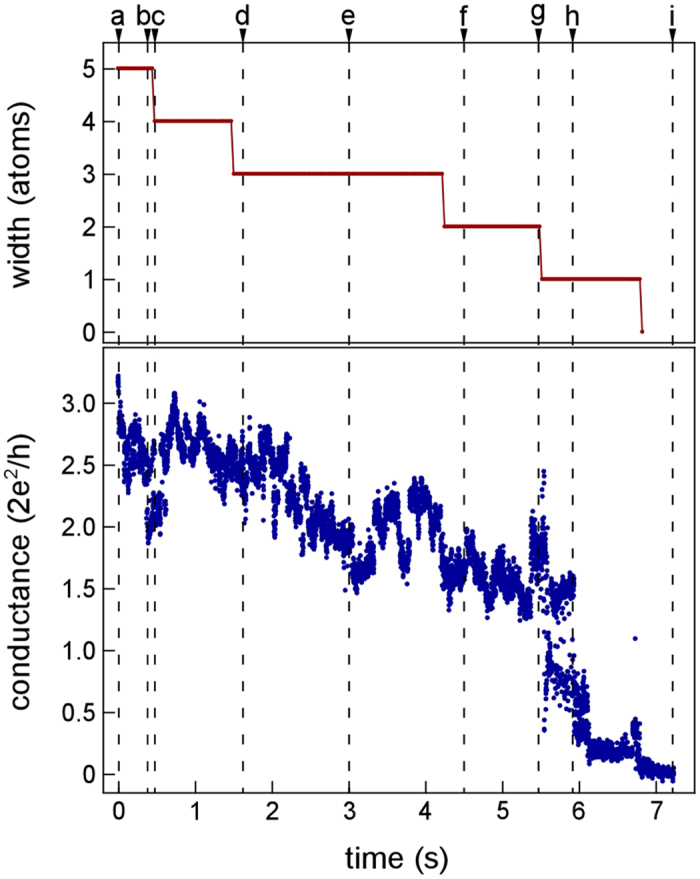
Variation in the minimum cross-sectional width and conductance of Zr NC as a function of time during the tensile deformation process shown in Fig. 1.

Figure 4 shows the variations in minimum cross-sectional width and conductance of the Zr NC during the tensile deformation process presented in Fig. 1 as a function of time. As the minimum cross-sectional width decreases, the conductance also decreases. The conductance increases rapidly at time g, as shown in Fig. 4. A similar increase in conductance is also observed between times h and i before contact fracture.

**Figure 5:**
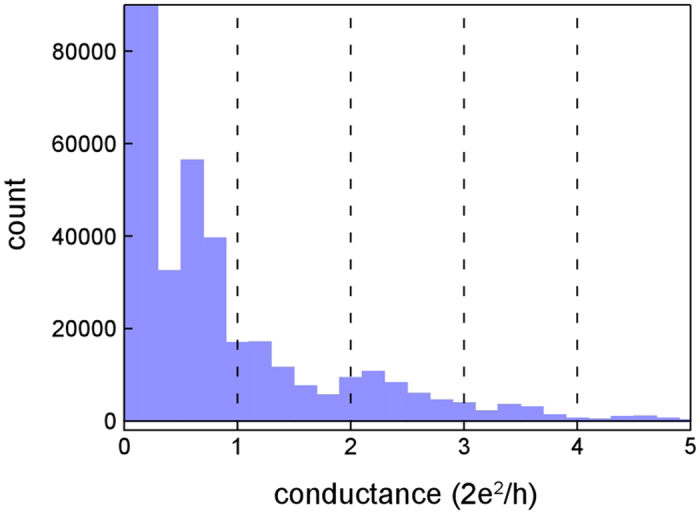
The conductance histogram obtained by repeated tensile deformation of Zr NCs. The applied bias voltage was 50 mV. The number of integrated traces was 90.

Figure 5 shows the histogram of time-conductance traces obtained during the tensile deformation processes of Zr NCs. The number of accumulated traces was 90. In the histogram, broad peaks are observed at 0.5G_0_ (G_0_ = 2*e*^2^/*h*, where *e* is the electron charge and *h* is Planck’s constant) and 1.8G_0_. These peaks do not correspond to the positions of integral multiples of G_0_. Thus, the features of the conductance quantization are not observed^27^,^28^,^29^,^30^,^31^,^32^,^33^,^34^.

**Figure 6:**
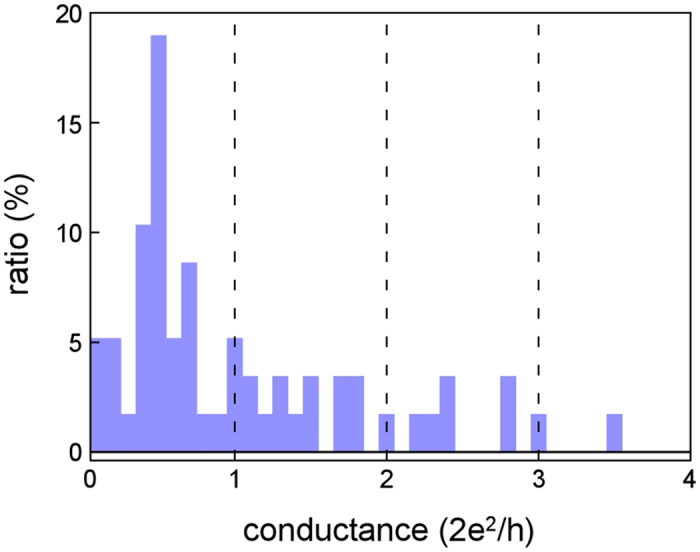
The ratio of the number of the plateaus at each conductance value to the number of plateaus observed in all conductance traces. We defined the plateaus as the duration of the part of a conductance trace falling within ± 0.05G_0_ variation in the duration of one imaging frame (66.7 ms).

Figure 6 shows the ratio of the number of plateaus at each conductance value to all observed plateaus. We defined the plateaus as the duration of the part of a conductance trace falling within ± 0.05G_0_ variation in the duration of one imaging frame (66.7 ms). Since the total number of observed plateaus is 90, the ratio of the 0.5G_0_ plateaus accounts for 19%.

**Figure 7:**
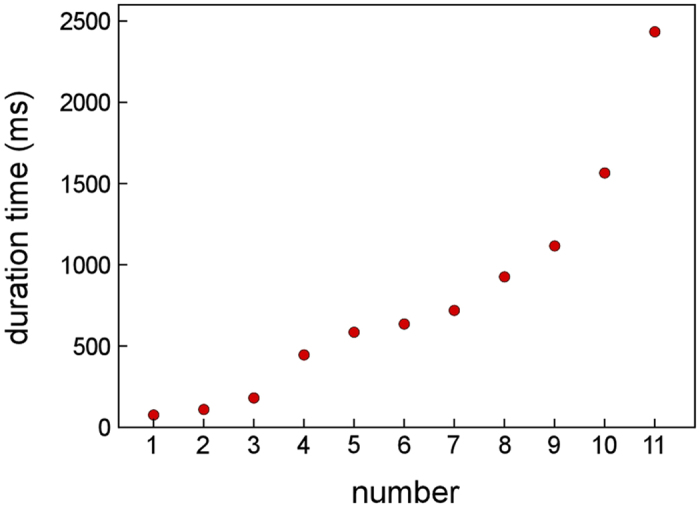
Duration for 0.5G_0_ plateaus. Data are sequenced in ascending order.

We next focused on the contact structures corresponding to the 0.5G_0_ plateaus. Figure 7 shows the duration time for the 0.5G_0_ plateaus. The number of 0.5G_0_ plateaus is 11 and the duration ranges from 70 to 2430 ms. Most of the plateau lengths of metallic NCs measured using mechanically controllable break junction methods and scanning tunneling microscopy are, at most, approximately 45 μs^17^,^27^,^28^,^29^,^35^,^36^,^37^,^38^. By contrast, the duration of plateaus defined in the present study is at least 1000 times longer than 45 μs; the present plateaus correspond to significantly stable structures.

**Figure 8:**
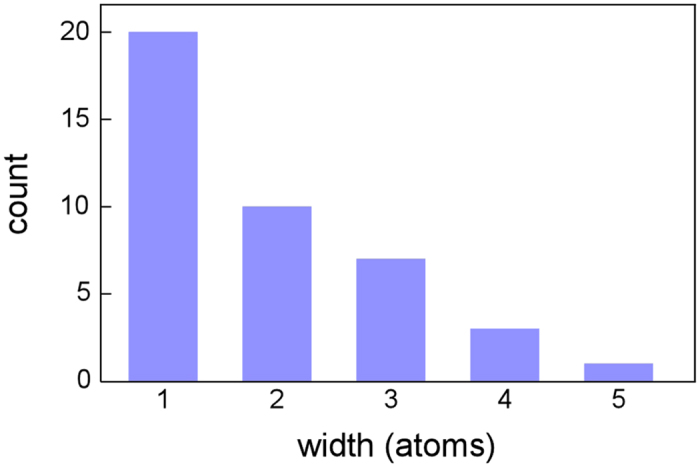
The number of Zr NCs exhibiting a conductance value of 0.5G_0_ against the minimum cross-sectional width.

Figure 8 shows the number of Zr NCs exhibiting a conductance value of 0.5G_0_ against the minimum cross-sectional width. The width ranges from one to five atom(s). The observation frequency decreases with the contact width. In particular, note that the ratio of one-atom-wide contacts accounts for 49% of all contacts.

## Discussion

### Suppression of slips and transformation to plastic flow deformation

For the NCs with fcc structure, e.g., gold (Au), Ag, and palladium (Pd), and that with an hcp structure, e.g., Zn, the tensile deformation occurs via slip along primary slip systems, i.e., {111}– < 110 > and (0001)–[2
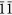
0], respectively^10^,^39^,^40^,^41^,^42^. In the tensile deformation process of the Zr NC observed in this study, slip first occurred on the (0001) boundary plane. This deformation is similar to that of Zn NCs with an hcp structure^42^. In a slip mechanism, the cycle of the accumulation of elastic strain and successive release by slip is repeated^41^,^43^. During this cycle, a relatively stable structure is formed after each release of strain. The lower the Young’s modulus of NCs, the higher the amount of elastic strain. The Young’s modulus of Au, Ag, and Pd with fcc structures and Zn with hcp structure is lower than 113 GPa, and that of Zr is comparable with these metals (96 GPa)^44^,^45^. Therefore, in the initial stage of the tensile deformation of the Zr NCs, we inferred that slip along the basal plane occurred. However, as this slip continues, as shown in Fig. 1(a)–(f), both nanotips separate, leading to slip suppression by the restoring force in the opposite direction of slip^11^. In addition, the contact width decreases to several atoms and slip without introduction of a dislocation occurs, i.e., homogeneous slip, resulting in the critical shear stress of theoretical slip^10^,^11^,^12^. As a result, slip is inhibited and Zr NCs begin to elongate toward the tensile force directions, which do not always correspond to primary and comparable slip systems. Thus, atoms are forced to move toward the tensile force direction rather than not along with slip mechanism, leading to deformation via breaking of interatomic bonds. Hence, it is inferred that such contacts were deformed in a plastic flow manner.

The deformation region observed in this study was smaller than several atom width and as a result, plastic flow deformation, which is different from well-known deformation observed in bulk states, such as slip, shear band in glass, and phase transformation^1^,^2^,^3^,^4^,^5^,^6^,^7^,^8^, was observed. Thus, the result shows that the well-known deformation mechanism is not activated due to size reduction to this size. In such small regions, no dislocation is introduced because if a dislocation is introduced in the region, elastic strain exceeds at least 20%, which leads to the destruction of the crystal structure^9^,^15^. Even if phase transformation occurs in the region, the transformed crystal structure can not be maintained due to high strain. In either case, it is difficult to consider that the well-known deformation mechanisms persist in the regions of several atom width. Homogeneous slip, in which no dislocation is mediated and the two regions beside a slip plane slide simultaneously, may occur if sufficient shear stress acts along primary or comparable slip systems^11^,^12^. The probability of the realization of this situation depends on the degree of slip in the crystal structure of the deformation region; for example, the number of primary and comparable slip systems. In particular, when a tensile force acts along the direction approximately perpendicular to the slip planes, as observed in Fig. 1, even no homogeneous slip is activated. As a result, it is deduced that none of the well-known deformation mechanisms, i.e., dislocation-mediated slip, phase transformation, or even homogeneous slip occur in the deformation regions smaller than several atom width, leading to the activation of other deformation mechanisms, in which the crystal structure get destroyed.

The atomistic behavior of deformation in metallic NCs have been investigated intensively by theoretical and computational methods in decades^9^,^15^,^16^,^17^,^18^,^19^,^20^,^21^,^22^,^23^,^24^,^25^,^26^,^32^,^46^,^47^,^48^,^49^. This is because there had been no experimental method to elucidate atomistic deformation dynamics of NCs since *in situ* transmission electron microscopy combined with piezomanipulation of specimens was developed^39^,^40^,^41^,^50^. Theoretical and computational studies show that whereas dislocation-mediated slip that occurs on one stress concentrated atomic plane is observed in NCs of widths larger than several nanometers, the atomic configuration of the deformation region becomes disordered and dislocation-mediated slip is no longer activated during the deformation in NCs with width less than several atoms; the deformation proceeds while the crystal structure crumbles and all atoms in the deformation region move simultaneously in a way like atomic motion in liquid^9^,^15^,^16^,^20^,^21^,^22^,^23^,^25^,^51^. Since the NCs does not melt during deformation, the deformation is expressed as plastic flow deformation in this study. Thus, the theoretical studies suggest that deformation mechanics transforms to non-slip manners when deformation regions are reduced to several atom width. Such simulations have been performed for NCs of fcc structure metals. As described above, hcp structure metals have fewer primary slip systems and slip is subjected to restriction in them in comparison with fcc metals^13^. Thus, it is also expected from the results of theoretical studies that non-slip deformation is prone to occur in NCs of hcp metals rather than in NCs of fcc metals.

### Relationship between stable structures formed by plastic flow deformation and aperiodic peaks in conductance histograms

In studies of NCs, the deformation including the variation in cross-sectional area has been discussed on the basis of the conductance measurements^34^. This is because the cross section of NCs has been estimated from the conductance values using the Sharvin formula or first-principle calculation^35^,^52^. In particular, the quantization of conductance observed in NCs, which suggests an orderly variation in the cross-sectional area of NCs during deformation, have been discussed in connection with cycles of elastic deformation and successive plastic deformation. Although an immense amount of study of NCs have been conducted, the relationship between structure and conductance of Zr NCs have not been investigated. The conductance histograms observed in Zr NCs (Figs 5, 6) showed an undulating distribution over a wide range with two broad peaks located in irregular interval. This feature does not correspond to the quantization of conductance, as observed in NCs of noble metals^29^, implying that the cross-sectional area of the Zr NCs does not change in a regular manner at an atomic scale.

The one- to five-atom-wide contacts before fracture were formed by plastic flow deformation. In Fig. 4, during the deformation, the observed conductance values were measured to be, at most, ~2G_0_ between times g and i. The conductance of the two peaks observed in the present study (0.5G_0_ and 1.8G_0_) are lower than this limit. Generally, peaks in conductance histograms imply the formation of stable structures. If deformation occurs via slip, the minimum cross-sectional area varies stepwise in a regular manner, which may lead to the emergence of periodic peaks in conductance histograms, e.g., features of the quantization of conductance that are observed in NCs of fcc metals^34^. However, during a plastic flow deformation process, no regularities in the variation of the minimum cross-sectional area and the atomic configuration are observed. Thus, as observed in this study, Zr NCs shows plastic flow deformation, in which the crystal structure crumbles and all atoms in the deformation region move simultaneously in a way like atomic motion in liquid. This leads to the formation of various electron paths and channels, resulting in non-quantified features of conductance. Thus, we inferred that the observed undulating distribution over a wide range with two broad peaks located in irregular interval in the conductance histogram observed in this study were attributed to irregular structural variation during plastic flow deformation.

The 0.5G_0_ peak was the highest, as observed in Figs 5, 6. We advance discussion of this peak. The peak emerges in the conductance histogram when contact structures having a certain value are maintained for longer times. When the structures corresponding to the 0.5G_0_ peak were observed, the deformation via slip was suppressed. The duration time of the 0.5G_0_ structures was recorded in a wide range (70–2430 ms)(Fig. 7) and the cross-sectional area ranged from one to five (Fig. 8). Thus, when the deformation via slip was suppressed, the structure was not identical and thus each structural variation was different. This is consistent with the results of TEM observation, i.e., the transformation to plastic flow deformation.

Metals that show the quantization of conductance are limited: s-electron metals, i.e., some of fcc structure noble metals, Au^17^,^20^,^27^,^29^,^31^,^36^,^37^,^38^,^53^,^54^,^55^,^56^, Ag^47^,^55^,^56^,^57^,^58^,^59^,^60^,^61^, copper^27^,^28^,^30^,^33^,^55^,^62^,^63^, and some of non d-electron metals and alkali metals, i.e., aluminum^64^,^65^,and sodium^27^. On the other hand, NCs of other metals including most of d-electron metals do not show the quantization features of the conductance. For example, in magnesium^66^, cobalt^67^, niobium^47^, tungsten^68^, and only one or a few broad peaks are observed in their conductance histograms. Zr is categorized into the latter and the present study confirmed this. Such non-quantization features of the conductance histograms may be attributed to irregular structural variation during plastic flow deformation.

### The effect of the strain rate and applied stress on the deformation

The average strain rate in this experiment was 1.4 × 10^−2^/s, which is 10^2^–10^3^ times larger than those in the deformation tests of nanometer-grained polycrystalline metals^2^,^7^. However, the strain rate equipped in an immense amount of contact-retraction experiments of NCs via mechanically controllable break junction methods and scanning probing methods is furthermore at least 100 times larger than that in this experiment^34^. In addition, the strain rate in MD simulations of tensile deformation of NCs is much higher than these values^9^,^25^,^51^. Even in such higher strain rates, slip is observed in both experiments and calculation. Thus, it is deduced that the strain rate in this experiment was not affected significantly on the transformation of deformation mechanism to non-slip mechanisms.

It is reported that when homogeneous slip occurs in metallic NCs, the yield stress increases to several gigapascals, corresponding to the theoretical strength of metals^10^,^11^,^12^. Although the yield stress acting on Zr NCs was not measured in this experiment, the stress of various NCs before fracture has been directly measured; the stress fluctuates drastically and the values exceed the yield stress of dislocation-mediated and homogeneous slip^10^,^41^,^60^. It is deduced that similar stress was applied to the Zr NCs before fracture. Thus, we inferred that the transformation of the deformation mechanism to plastic flow manner observed in this study is attributed to the formation of disordered structures via the elongation along forbidden directions for slip due to the decrease in the number of effective slip systems in the hcp structure under such tensile stress.

## Conclusion

We observed the tensile deformation process of Zr NCs by *in situ* TEM and investigated the relationship between their structures and conductance. We found that Zr contacts deformed in a plastic flow manner, which differed from slip mechanisms, when the contact size decreased to less than several atoms. Irregular variation in the minimum cross-sectional area and atomic configuration of the contact region was observed during plastic flow deformation. We inferred that plastic flow deformation contributed to the emergence of aperiodic broad peaks at 0.5G_0_ and 1.8G_0_ in the conductance histogram.

## Methods

We used *in situ* TEM combined with a conductance measurement system using piezo-manipulation of two nanotips^30^. We prepared two Zr nanotips via mechanical polishing and argon-ion milling; the nanotips were in the form rectangular plates having 10-nm-thick edges. We manipulated the nanotips using piezo-manipulation inside a transmission electron microscope to produce NCs. Subsequently, the NCs became elongated when a bias voltage of 50 mV was applied. We performed a series of these operations at room temperature in a 10^–5^ Pa vacuum. We observed the structural variation using high-resolution TEM lattice imaging with a charge-coupled-device camera and measured the conductance using a two-terminal method. High-resolution imaging frames were obtained at intervals of 66.7 ms.

## Additional Information

**How to cite this article**: Yamada, K. and Kizuka, T. Transformation from slip to plastic flow deformation mechanism during tensile deformation of zirconium nanocontacts. *Sci. Rep.* **7**, 42901; doi: 10.1038/srep42901 (2017).

**Publisher's note:** Springer Nature remains neutral with regard to jurisdictional claims in published maps and institutional affiliations.

## Supplementary Material

Supplementary Information

Supplementary Movie 1
