# Supplementary material for: Transformation from slip to plastic flow deformation mechanism during tensile deformation of zirconium nanocontacts
Source: Sci Rep. 2017 Feb 20;7:42901. doi: 10.1038/srep42901 (PMC5317169; doi:10.1038/srep42901)
Supplement: Supplementary Information [file srep42901-s1.pdf]

Supplementary Information

Title: Transformation from slip to plastic flow deformation mechanism during tensile deformation of zirconium nanocontacts

Authors: Kohei Yamada and Tokushi Kizuka

The Supplementary Information is the movie corresponding to Fig. 1. The movie caption is as follows.

Movie caption

Movie 1 | Movie of *in situ* high-resolution TEM of tensile deformation of a Zr NC, corresponding to Fig. 1.
